# Medical Uses of the Telephone

**Published:** 1878-07

**Authors:** 


Medical Uses of the Telephone.—We have already recorded various experiments and suggestions with reference to the medical uses of the telephone. It has been in the house of a medical man during the last few weeks, to enable a member of the family suffering from an infectious exanthem, to communicate with her family and friends, and this application we would recommend as very practical to the managers of fever-hospitals and asylums. In the *Boston Medical and Surgical Journal* we read that its utility in the class demonstration of auscultative signs of disorder of the chest, is being studied, with good promise of success. Prof. DaCosta made a preliminary trial in March last, at the Pennsylvania Hospital, of a Bell's telephone, constructed by Dr. W. B. Hopkins, a former resident. It was tested by cases of cardiac murmurs and different varieties of respiration, and, while the results obtained were not fully satisfactory, it was believed to be demonstrated that a slight modification in the construction of the instrument, enabling it to respond to more delicate impulses, would fit it for the purpose, and make it an almost indispensable adjunct to the clinical amphitheatre.—*Hospital Gazette.*

---

Dr. S. S. Smith, of Driftwood, Pa., writes to the *Medical and Surgical Reporter* that he recently delivered a woman of a child weighing twenty-two pounds. He states that it was necessary to first perform craniotomy, which statement will perhaps enable the more credulous of our readers to swallow the story.—*Michigan Medical News.*

